# Pilot Study of a Mobile, Virtual Reality–Based Digital Therapeutic for Smoking Cessation: Randomized Controlled Trial

**DOI:** 10.2196/66411

**Published:** 2025-11-12

**Authors:** Yeong-Seon Jo, Arom Pyeon, Min-Kyung Hu, SungMin Kim, In-Young Choi, Dai-Jin Kim, Ji-Won Chun

**Affiliations:** 1 Department of Medical Science Seoul St. Mary’s Hospital The Catholic University of Korea College of Medicine Seoul Republic of Korea; 2 Department of Medical Informatics Seoul St. Mary’s Hospital The Catholic University of Korea College of Medicine Seoul Republic of Korea; 3 Catholic Medical Center Institute for Basic Medical Science The Catholic University of Korea Seoul Republic of Korea

**Keywords:** digital therapeutics, virtual reality, VR, mobile health, mHealth, smoking cessation, nicotine dependence, cognitive behavioral therapy, CBT, motivational enhancement therapy, MET, randomized controlled trial, RCT

## Abstract

**Background:**

Smoking cessation remains a global challenge, with traditional treatments showing limited long-term success due to low adherence and accessibility issues. Digital therapeutics, such as mobile apps and virtual reality (VR)–based interventions, could offer innovative solutions for smoking cessation treatment. NICO-THERA is a digital therapeutic program integrating cognitive behavioral therapy (CBT) and motivational enhancement therapy (MET) to address nicotine dependence.

**Objective:**

This study aims to evaluate the safety and efficacy of NICO-THERA, a digital therapeutic intervention combining VR and mobile apps, for supporting smoking cessation among individuals with nicotine dependence. The primary focus was on smoking abstinence, nicotine dependence reduction, and motivation to quit smoking over a 12-week intervention period.

**Methods:**

This open-label, exploratory, randomized controlled trial involved 30 participants randomly assigned to the digital therapeutic group (DTG; n=15; mean age 43.1 years; mean number of daily cigarettes: 9.5) or basic treatment group (BTG; n=15; mean age 48.7 years; mean number of daily cigarettes: 9.2). The DTG received the NICO-THERA program, involving VR sessions (relaxation training, craving coping, and refusal skills) and mobile app–based CBT and MET modules for daily therapeutic exercises. The BTG received basic care including video education and printed materials. The primary outcomes were the 7-day point prevalence abstinence (PPA) and 30-day PPA at 8 weeks and 12 weeks. Secondary outcomes included nicotine dependence (Fagerström Test for Nicotine Dependence [FTND]) and motivation to change (Stages of Change Readiness and Treatment Eagerness Scale–Smoking [SOCRATES-S]).

**Results:**

There were no significant differences in 7-day and 30-day PPA between the DTG and BTG. However, in within-group analyses, the DTG showed significant improvements in both 7-day and 30-day PPA at week 8 (*z*=–2.00, *P*=.046), along with consistent reductions in smoking days and cigarette consumption across all time points. The BTG only significantly decreased cigarette consumption at week 8. Additionally, the DTG exhibited a significant increase in taking steps of motivation at week 12 (*U*=19.00, *P*=.048) compared with the BTG. No adverse device effects were reported. Adherence to smoking cessation diaries and medication logs was higher in the DTG (mean 83 of 84 days, 99%; 9 participants) than in the BTG (mean 74 of 84 days, 88%; 12 participants), based on the 12-week average adherence among those who completed the study.

**Conclusions:**

The NICO-THERA digital therapeutic program demonstrated preliminary effectiveness at reducing nicotine dependence. Additionally, participants in the DTG group exhibited a progressive improvement in their motivation to quit smoking, as reflected by a significant reduction in ambivalence at week 8 and increase in proactive cessation efforts at week 12. These results suggest that the structured integration of MET and CBT within the NICO-THERA program effectively reinforced psychological readiness for smoking cessation, fostering a sustained commitment to behavioral change.

**Trial Registration:**

Clinical Research Information Service, Republic of Korea KCT0009801; https://cris.nih.go.kr/cris/search/detailSearch.do?seq=28285&status=5&seq_group=28285&search_page=M

## Introduction

The global tobacco crisis continues to be one of the most severe public health challenges, with more than 8 million fatalities annually worldwide [1], of which 6.2 million deaths are directly linked to tobacco consumption and approximately 1.3 million are owing to exposure to second-hand smoke among nonsmokers [2]. According to the 2023 Organisation for Economic Co-operation and Development (OECD) Health Statistics, the average smoking rate across OECD countries is 15.9%, with South Korea showing a comparable rate of 15.4% [3]. Tobacco use is a leading contributor to a multitude of health issues, including lung cancer, heart disease, and chronic respiratory conditions, resulting in significant social and economic repercussions [4]. Although South Korea has implemented comprehensive smoking cessation policies and invested substantially in public health initiatives [5], as the variety of tobacco products increases, the willingness of smokers to quit smoking has been under threat [6]. These trends indicate that smoking cessation policies must evolve to address the changing landscape of tobacco use and cessation efforts.

Despite the known harm of smoking, many smokers find quitting to be a challenging endeavor. Characterized as a chronic relapsing condition [7], smoking cessation frequently leads to a range of withdrawal symptoms such as cravings, irritability, depression, and restlessness [8]. Notably, the success rate of individuals attempting to quit smoking unaided without professional support remains low, with estimates ranging from 3% to 5% [9]. Medications are designed to mitigate the discomfort associated with nicotine withdrawal symptoms, thereby significantly enhancing the likelihood of smoking cessation [10,11]. The most effective smoking cessation strategy appears to be a combination of behavioral therapy and pharmacotherapy [10-13], a method widely supported by national smoking cessation programs across various countries [14-19].

To overcome the limitations of accessibility identified in traditional smoking cessation treatments and provide cost-effective and time-efficient interventions, the development of digital therapeutics (DTx) for smoking cessation has been extensively pursued. Mobile health (mHealth) technologies for smoking cessation began as basic text message–based interventions. Although effective [20-23], these early digital tools had limited functionality [21,24]. Over time, these have evolved into complex smartphone apps and other forms of DTx that use technology to offer more accessible, flexible, and personalized support [25-28]. DTx are characterized as interventions based on evidence provided by certified software programs to prevent, manage, or treat medical conditions, separating them from conventional wellness products [29]. Following the development of wellness apps for smoking cessation, some DTx specifically designed for smoking cessation have incorporated behavioral strategies such as cognitive behavioral therapy (CBT) to support cessation efforts, while others have focused on alternative behavioral modification techniques [30-34]. The efficacy of DTx devices has also been confirmed in studies with small sample sizes [35].

In addition to smartphone-based interventions, encouraging advances have been made in the use of virtual reality (VR) in digital medicine. VR has been implemented in consultations and hospital settings under the supervision of health professionals [36]. Incorporating VR into treatments can alleviate patient symptoms in conditions such as claustrophobia [37,38], post-traumatic stress disorder [39], and smoking cessation [40,41] and may improve adherence to weight loss behaviors [42]. Expanding the role of VR in smoking cessation, recent studies have explored its potential to not only assist with reducing cravings and withdrawal symptoms but also modify behavioral patterns and increase long-term abstinence rates. For instance, the cue exposure therapy approach, which uses VR to simulate real-life smoking triggers in a controlled environment, has demonstrated promising results for helping individuals resist cravings by practicing coping strategies in virtual scenarios [40]. Similarly, immersive VR environments have been used for mindfulness-based interventions to reduce stress and anxiety associated with quitting smoking, thereby supporting overall mental well-being during cessation efforts [41]. 

Building on recent advances in digital-based smoking cessation treatments, such as mobile apps and VR, we developed NICO-THERA, a unified DTx that integrates both a mobile app and VR to promote smoking cessation. Although some prior studies have combined VR with therapeutic components—such as cue exposure therapy or mindfulness-based approaches [40,41]—these were typically delivered alongside CBT or as independent modules, rather than through an integrated, week-by-week structure. In contrast, NICO-THERA incorporates CBT and motivational enhancement therapy (MET) techniques into a structured digital program in which VR sessions are systematically aligned with weekly cognitive-behavioral content. This study examined the safety and efficacy of NICO-THERA as a comprehensive, multimodal intervention, rather than relying on standalone VR or mobile app treatments.

## Methods

### Study Design

This study was conducted as a 2-arm, open-label, exploratory, randomized controlled clinical trial (RCT). The primary objectives were to evaluate the preliminary efficacy and safety of NICO-THERA for smoking cessation. Outcomes were assessed directly by the researchers without independent evaluators. Although the absence of blinding may introduce potential biases, the focus of this study was on generating initial data rather than establishing definitive efficacy conclusions.

Participants were randomly assigned to either the digital therapeutic group (DTG) or the basic treatment group (BTG) using a pregenerated 1:1 random allocation sequence. Each participant was assigned to a group according to their screening number in the order of arrival. The allocation sequence was predetermined for screening numbers 1 to 30, ensuring that neither the participants nor the researchers knew in advance which group the participants would be allocated to, thereby maintaining the randomness of the allocation process. According to guidelines for pilot trials, a minimum sample size of 12 participants per treatment arm was recommended to ensure meaningful preliminary analysis [43,44]. Additionally, small-sample RCT studies, including those related to smoking cessation, have been conducted with a minimum of 9 and a maximum of 19 participants per group. [45-48]. In this study, 15 participants were enrolled in each group (DTG and BTG) to account for potential attrition, resulting in a total enrollment of 30 individuals. No significant changes in the trial methodology were made after the trial commenced.

We used the Simulator Sickness Questionnaire (SSQ), administering it before and after each VR session to monitor potential adverse effects and evaluate safety in DTG. To indicate preliminary efficacy, we measured 7-day and 30-day point prevalence abstinence (PPA) rates as primary outcomes, as well as changes in nicotine dependence and motivation to quit smoking as secondary outcomes. Additionally, pharmacological therapy and participation in outpatient counseling were monitored through clinical records.

**Participants and Recruitment**

The study population consisted of patients visiting Seoul St. Mary’s Hospital for smoking cessation and those who responded to a clinical study recruitment advertisement. Written informed consent was obtained from all participants before eligibility screening. Medical staff evaluated the participants based on the inclusion and exclusion criteria through individual interviews. Data were collected in a clinical setting at Seoul St. Mary’s Hospital, where the participants attended outpatient visits. Screening and follow-up assessments were performed in clinical settings. Participants who met the eligibility criteria were randomly assigned to either the DTG or BTG. Regardless of the assigned group, all participants received usual care for smoking cessation, which included counseling from medical staff at every visit as well as personalized pharmacological therapy with varenicline or bupropion. Of the 21 participants, 14 (including 7 in the DTG) began pharmacotherapy at baseline, while the remaining 7 (including 2 in the DTG) initiated pharmacotherapy later in the study. The in-treatment period commenced with randomization and lasted until week 12, during which follow-up visits were conducted at 4-week intervals (weeks 4, 8, and 12).

The inclusion criteria were as follows: (1) adults who were at least 19 years old and younger than 75 years; (2) individuals diagnosed with nicotine addiction according to the clinical criteria for mental and behavioral disorders owing to use of tobacco (F17) or toxic effect of tobacco and nicotine (T652) as per the Korean Classification of Diseases and in accordance with the clinical criteria for nicotine use disorders specified in the Diagnostic and Statistical Manual of Mental Disorders, Fifth Edition (DSM-5) [49]; (3) individuals who smoked an average of 5 or more cigarettes per day in the past 6 months; (4) participants who were able to communicate with the researcher and consented to the procedures required by the study protocol, understanding the objectives of the research and signing the consent form; (5) smartphone users who agreed to install and use an app for data collection and management; and (6) smartphone users who agreed to receive text messages and respond to survey items via their smartphones, consenting to data collection and management.

The exclusion criteria included (1) individuals who did not meet the clinical criteria for mental and behavioral disorders owing to use of tobacco (F17) or toxic effect of tobacco and nicotine (T652) according to the Korean Classification of Diseases, based on individual interviews with a specialist or researcher; (2) individuals with cognitive impairments affecting decision-making capacity; (3) individuals with logistical or personal constraints preventing full participation in the study, including scheduled visits and intervention activities (eg, long-term travel plans, conflicting work schedules, or severe mobility limitations); (4) those who had started or were undergoing another CBT within the last 3 months; (5) individuals who either did not own a smartphone registered in their name or lacked sufficient ability to use a smartphone for study-related tasks, such as receiving text messages, completing self-report logs, and engaging with app-based interventions; (6) individuals without a smartphone or significantly limited ability to use smartphones including receiving text messages; (7) those at severe risk of depression, suicidal ideation, or suicide attempts; and (8) other reasons deemed by the investigator as inappropriate for participation in the clinical trial. The participants completed eligibility screening and provided informed consent.

### Intervention Condition

#### DTG

**Figure 1:**
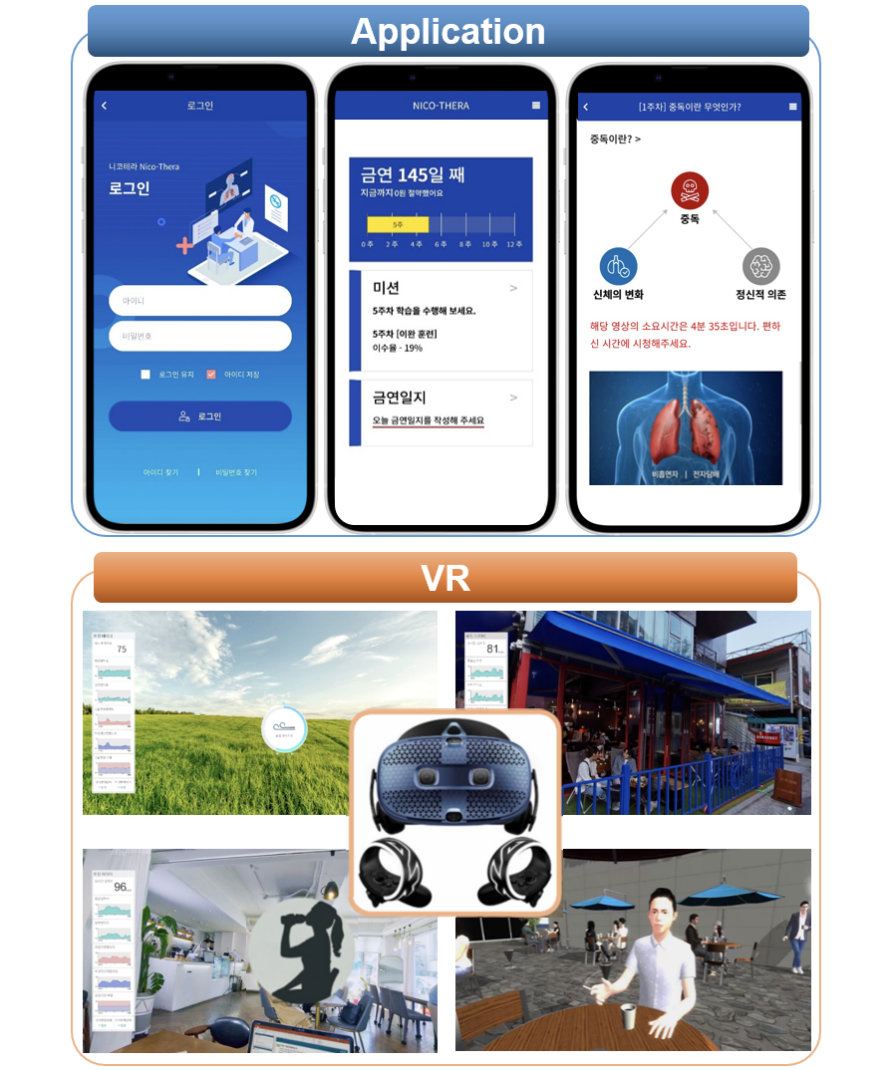
Overview of the NICO-THERA digital therapeutic program, including app screenshots of the main dashboard and cognitive behavioral training (CBT)-based contents and virtual reality (VR)-based sessions for relaxation training, craving coping, and refusal training.

NICO-THERA, the DTx used in this study, is software as a medical device designed to treat and manage nicotine dependence. NICO-THERA has been classified as a Class 2 (low-risk) medical device for cognitive therapy software by the Ministry of Food and Drug Safety in South Korea (E066060.02). Figure 1 illustrates the interface elements of the NICO-THERA, including screenshots of its main features. The app’s content was reviewed by clinical experts in smoking cessation and CBT to ensure the accuracy and effectiveness of the therapeutic modules. The NICO-THERA app was developed through several iterations, including usability testing with target users, to refine the interface and content based on feedback. The intervention for the DTG was a structured 12-week program consisting of 2 key components: VR therapy content provided via a head-mounted display and a smartphone app. This nicotine DTx content is based on CBT and MET, which are prominent evidence-based therapies aimed at improving addictive disorders. The NICO-THERA app and VR content used in this study were all from Version 1.0, ensuring consistent therapeutic content with no updates nor modifications throughout the trial.

The VR content was delivered using the HTC Vive Cosmos headset, which includes integrated headphones for immersive audio output. VR sessions were conducted exclusively in the hospital at designated PC stations, ensuring a controlled and consistent environment. Participants underwent 3 VR sessions at weeks 0, 4, and 8 during their clinic visits, with each session lasting approximately 10 minutes. Each VR session focused on a specific module, progressing sequentially throughout the intervention period: Image Relaxation Training at week 0, Craving Coping Training at week 4, and Refusal Training at week 8. Image relaxation training, rooted in behavior modification theory, helps participants cope with stress-related triggers [50,51] such as depression and anxiety by practicing deep breathing and relaxation techniques within a VR environment that replicates real-life settings. Participants can choose a preferred virtual setting among the forest, at a campfire, or on the beach to conduct the imagery relaxation training (Multimedia Appendix 1). Craving coping training (Multimedia Appendix 2) uses role-playing in a realistic VR setting to apply learned relaxation and behavioral strategies to manage situations that trigger smoking cravings [52,53]. Finally, refusal training (Multimedia Appendix 3) focuses on strengthening the participants’ ability to clearly refuse smoking offers by practicing assertive communication and refusal skills [54] in VR scenarios that mirror real-life interactions, thereby bolstering their smoking cessation efforts. Participants interacted with the VR environment using handheld controllers to navigate scenarios and respond to prompts, such as selecting preferred settings (eg, forest, campfire, or beach) or engaging in role-playing scenarios. In addition, although VR sessions were separate from the smartphone app, data from participant responses in the VR session were synchronized with the smartphone app for program monitoring.

The participants accessed the NICO-THERA app on their smartphones. The app was provided free of charge, and the participants were given detailed instructions on how to download and install it. The app required an internet connection for certain features, and participants were encouraged to maintain access to the internet throughout the study.

The NICO-THERA app integrates CBT and MET to deliver structured therapeutic interventions tailored to nicotine addiction. According to the stages of change model, intentional behavior change, such as quitting smoking, typically progresses through 5 stages: Precontemplation, Contemplation, Preparation, Action, and Maintenance [55-57]. NICO-THERA is a 12-week program that aligns with the 5 stages of change. It is also designed to support smoking cessation and maintenance by integrating evidence-based educational, cognitive, behavioral, and emotional techniques derived from MET and CBT [30,58]. This app is designed to facilitate users’ engagement in their treatment processes by providing therapeutic sessions that help identify triggers, manage cravings, and develop effective coping strategies. Each of the 12 weekly topics was released sequentially, with one topic becoming available each week to align with the structured program. Participants were allowed to review past topics multiple times but could not access future topics ahead of schedule. This comprehensive approach encompasses operant conditioning to modify behaviors associated with smoking, cognitive restructuring to address dysfunctional thoughts related to nicotine use, and relaxation training to manage stress and emotional triggers. Specifically, the initial 1 to 4 weeks of the program emphasized MET approaches, which are effective for promoting behavioral changes for smoking cessation. From week 5 onward, the program gradually introduced cognitive, behavioral, and emotional techniques focused on CBT, helping individuals acquire strategies that are practically useful for maintaining smoking cessation. Each lesson took approximately 10 minutes per day to complete. Upon completion of the content each week, a quiz was provided to evaluate the effectiveness of the weekly program and reinforce the users’ knowledge. The weekly topics and contents are detailed in Table 1.

In addition to these core therapeutic strategies, the app is equipped with several practical tools to support patients and health care providers. It includes a smoking cessation diary and medication adherence log, which are instrumental for tracking daily smoking behaviors and medication intake. Participants were instructed to record their smoking cessation diary and medication adherence log daily and to complete the weekly lessons provided through the app. Each lesson took approximately 10 minutes per day to complete. These features allow health care providers to monitor the patient’s progress and compliance more effectively during clinical visits, providing crucial data that can be used to adjust treatment plans and interventions accordingly (Multimedia Appendix 4). The participants received weekly push notifications from the app reminding them to complete their therapy sessions and log their smoking behaviors.

**Table 1:** Cognitive behavioral therapy (CBT) and motivational enhancement therapy (MET) techniques included in the NICO-THERA app.

Time point	Weekly program topics	Detailed content
Registration	Understanding Application Usage	Familiarization with the application’s functionalities; completion of registration and provide basic information
Week 1	Treatment Plan and Functional Analysis	Structured approach for addiction treatment and identification of users’ nicotine usage patterns
Week 2	Motivation Enhancement Training	Education on motivational theories, change motivations, and stages of change
Week 3	Creating an Environment for Success	Understanding conditioning and association and eliminating factors related to smoking
Week 4	Identifying Triggers and Cravings	Pinpointing specific smoking triggers and understanding the underlying mechanisms of cravings
Week 5	Relaxation Training	Education and implementation of relaxation training with biofeedback signals
Week 6	Behavioral Craving Coping Strategies	Understanding cravings and learning strategies to cope with them
Week 7	Identifying Cognitive Distortions	Recognizing and categorizing thought patterns that contribute to smoking behavior
Week 8	Responding to Cognitive Distortions	Developing strategies to challenge and reframe distorted thoughts
Week 9	Refusal Training	Learning assertive communication skills useful in smoking cessation
Week 10	Coping with Depression and Anxiety	Learning about and dealing with depression and anxiety
Week 11	Coping with Anger	Learning about and dealing with anger
Week 12	Relapse Prevention and Review	Recognizing the possibility of relapse and responding to warning signs

#### BTG

In the control group, participants received both video and printed educational materials as part of their treatment for nicotine addiction. Specifically, video education comprised CBT-focused content tailored to manage nicotine addiction. We provided 3 videos during the study: the first video at baseline (week 0), the second at week 4, and the third at week 8. Each video introduced distinct strategies for managing nicotine addiction, aligned with the participants’ progress in the study. Additionally, during their 12-week outpatient visits, they were provided with standardized educational booklets on CBT and MET prepared by the Ministry of Health and Welfare of the Republic of Korea. The first booklet (weeks 0-3) focused on the harmful effects of smoking and the mechanisms of nicotine dependence, the second (weeks 4-7) addressed coping strategies for withdrawal symptoms and cravings and offered relapse prevention techniques, and the third (weeks 8-11) emphasized strategies for sustaining abstinence and managing stress and negative emotions. Printed materials, including educational booklets, smoking cessation diaries, and medication logs, were provided at baseline (week 0), week 4, and week 8. The smoking cessation diaries and medication logs were collected at the subsequent visits (weeks 4, 8, and 12) to monitor participants’ adherence and progress. These resources were designed to improve the understanding and handling of nicotine-use disorders.

### Assessments and Outcomes

Prior to the commencement of the intervention, participants completed a comprehensive baseline questionnaire designed to collect demographic information and detailed smoking history. Demographic information included age, sex, education level, past and current medical histories, and information on concurrent medications. The questionnaire also inquired about participants’ smoking history, including the type of tobacco products, number of cigarettes consumed per day, age at initiation, previous attempts at quitting, and methods previously used in those attempts.

For the primary efficacy indicators of smoking cessation maintenance, assessments were conducted at 2 time points: during treatment at weeks 8 and 12. At each of these time points, both the 7-day and 30-day PPA were assessed. Self-reported smoking abstinence was determined by asking patients whether they had smoked one or more cigarettes in the last 7 and 30 days. All assessments were conducted through verbal self-reports in clinical interviews with medical staff. Additionally, to enhance the validity of these self-reports, a qualitative saliva cotinine test and an exhaled carbon monoxide test, a recognized biological marker of nicotine exposure, were concurrently administered. If the results of these biological tests contradicted the self-reported abstinence, the participants were considered to have failed to maintain smoking cessation.

Secondary efficacy indicators (supplementary indicators) included the Fagerström Test for Nicotine Dependence (FTND) [59], a widely used instrument for evaluating the severity of nicotine addiction. The FTND consists of 6 questions that assess various aspects of nicotine dependence, including the time to the first cigarette after waking up, difficulty of refraining from smoking in nonsmoking areas, and number of cigarettes smoked per day. Each question is scored on a scale, with higher total scores indicating greater nicotine dependence. Additionally, the Stages of Change Readiness and Treatment Eagerness Scale-Smoking (SOCRATES-S) was used to measure smokers’ motivational readiness [60]. It assesses 3 key dimensions of motivation: recognition, ambivalence, and the steps taken. The recognition reflects a smoker’s acknowledgment of the problem and the understanding that smoking is detrimental to health. Ambivalence measures the smoker’s mixed feelings about quitting, capturing the internal conflict between the desire to quit and fear of change. Taking steps evaluates the proactive efforts that the smoker has already made toward cessation. Each item is scored on a 5-point Likert scale, ranging from “strongly disagree” (1 point) to “strongly agree” (5 points). Subscale scores are calculated by summing the scores of the individual items within each dimension, providing a range of possible scores for each subscale. For example, higher recognition scores indicate a stronger acknowledgment of the problem, while higher ambivalence scores reflect greater internal conflict. Higher scores in the taking steps dimension signify more active efforts toward cessation. The SOCRATES-S score provides insight into where smokers are on the continuum of change, ranging from precontemplation to maintenance, thereby guiding individualized treatment approaches.

Safety assessments for the VR intervention were conducted using the SSQ [61], which evaluates potential adverse effects such as nausea or disorientation, ensuring participant safety in the VR components of the study. The SSQ was administered twice, before and after each VR session, to monitor any changes in symptoms. The SSQ includes 16 symptoms, each rated on a 4-point Likert scale (0=none, 1=slight, 2=moderate, 3=severe). The scores are aggregated into 3 subscales (nausea, oculomotor, and disorientation) and a total severity score using weighted formulas. A cutoff score of 20 points was used in our study; if this threshold was exceeded, VR sessions would have been discontinued, and an adverse event report would have been filed in accordance with our protocol [62,63]. This protocol was established to prioritize participant safety throughout the study.

### Data Analysis

In this study, intention-to-treat (ITT) analysis was primarily used to evaluate the primary efficacy outcomes. For the ITT analysis, a total of 30 participants were included, with 15 participants in the DTG group and 15 participants in the BTG group. To handle missing data, the last observation carried forward method was used. However, as the majority of participant dropouts occurred prior to the first follow-up visit (week 4) after enrollment, a per-protocol (PP) analysis was additionally conducted for the secondary efficacy outcomes to assess the treatment effects among those who adhered to the intervention. The PP analysis included only participants who completed the 12-week study protocol, excluding those who dropped out. Specifically, data from participants with complete records at all time points were analyzed, resulting in a total of 21 participants: 9 from the DTG and 12 from the BTG. All statistical analyses were performed using SPSS version 28 (IBM Corp). Descriptive statistics were used to examine the demographic characteristics and smoking or quit-related attributes of participants. Subsequently, at baseline and during treatment weeks 4, 8, and 12, the mean values of efficacy indicators, nicotine use assessments, and motivation to change indicators were compared between the DTG and the BTG to evaluate changes over time. This exploratory clinical study had a small sample size, with only 15 participants assigned to each group. Normality tests indicated that the data did not follow a normal distribution (*P*<.05), thereby invalidating the assumption of normality. Consequently, nonparametric tests that are appropriate for small sample sizes and do not require the assumption of normality were used [64]. Specifically, statistical significance was assessed using the Wilcoxon signed rank test and the Mann-Whitney *U* test, which analyzes rank-based data [65,66].

### Ethical Considerations

This study was conducted according to the ethical guidelines outlined in the Declaration of Helsinki. Ethical approval for the study protocol was obtained from the Ministry of Food and Drug Safety (number 1271) and the Institutional Review Board of Seoul St. Mary’s Hospital (KC21DNSS0706). All participants provided written informed consent after being thoroughly informed about the study’s objectives, procedures, risks, and benefits. Participant data were anonymized and securely stored to ensure confidentiality. Identifiable information was removed from the dataset, and only deidentified data were used for analysis. Data access was restricted to authorized researchers. Participants were compensated ₩40,000 (US $28.69) per visit for their participation, amounting to a total of ₩160,000 (US $114.76) for completing the 4 study visits. This compensation was intended to cover transportation and time-related costs and was not contingent upon their smoking cessation outcomes.

## Results

### Participant Engagement and Safety Outcomes

**Figure 2:**
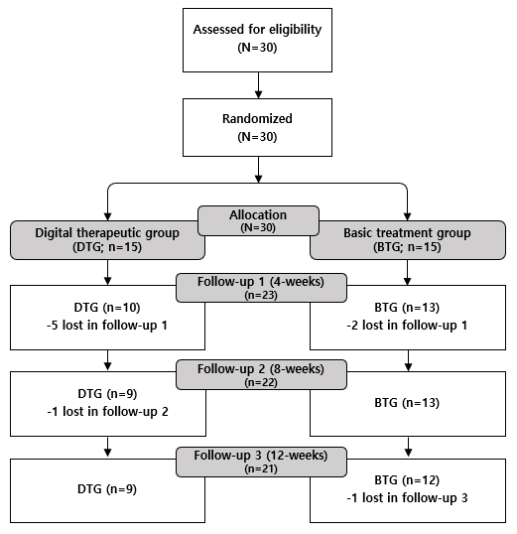
Study participant CONSORT (Consolidated Standards of Reporting Trials) flow diagram.

A CONSORT flow diagram is depicted in Figure 2. In this study, 30 participants were randomly assigned to either the DTG (15 participants) or the BTG (15 participants). Both groups completed the baseline visit at week 0 (visit 2). By the fourth week (visit 3), 7 participants had dropped out (5 from the DTG and 2 from the BTG), leaving 23 participants in the study follow-up period (10 in the DTG and 13 in the BTG). By week 8 (visit 4), 1 additional participant from the DTG had dropped out, resulting in 22 participants remaining in the study (9 in the DTG and 13 in the BTG). By the 12th week (visit 5), 1 more participant from the BTG had dropped out, and a total of 21 participants (9 in the DTG and 12 in the BTG) successfully completed the study follow-up period. The overall attrition rate was 30% (9/30), with 40% (6/15) in the DTG and 20% (3/15) in the BTG. The adherence rates for maintaining a smoking cessation diary and medication log were 99% (mean 83 of 84 days) in the DTG (12-week average diary and log adherence rate of 9 participants) and 88% (mean 74 of 84 days) in the BTG (12-week average diary and log adherence rate of 12 participants). These adherence rates were calculated by dividing the number of diary and log entries each participant recorded over the 12-week (84-day) intervention period by the total expected number of entries then averaging these individual adherence rates across participants in each group. This indicates higher treatment adherence in the DTG group. Upon reviewing the DTG’s app usage monitoring, the average completion rate of the app-based treatment program was 91% (11 weekly modules of the 12 weeks of modules; 9 participants), while the average achievement rate of the app treatment program was 98% (12-week average quiz achievement rate by week for 9 participants) based on the proportion of completed quizzes relative to the number of accessed modules).

In this exploratory clinical trial, the SSQ was used to assess the symptoms of motion sickness induced by a virtual environment before and after the use of a VR device. The results indicated that neither the pre- nor postuse scores exceeded the cutoff of 20 and no significant differences were observed between the 2 scores. Additionally, no adverse events related to the use of the VR or smartphone app were reported, confirming the safety of the DTx software NICO-THERA.

### Participant Characteristics at Baseline

The demographic characteristics of the participants are presented in Table 2. The mean ages were 43.07 (SD 12.15) years in the DTG and 48.67 (SD 14.53) years in the BTG. Of the 15 participants in the DTG, there were 11 (73%) men and 4 (27%) women, whereas of the 15 participants in the BTG, there were 12 (80%) men and 3 (20%) women. Regarding educational attainment, the highest proportion of participants (9/15, 60%) had completed a bachelor’s degree in both the DTG and BTG.

The average length of time participants had been smoking was 23.32 (SD 11.22) years in the DTG and 29.21 (SD 12.90) years in the BTG. Daily cigarette consumption was similar, with DTG participants smoking an average of 9.53 (SD 8.16) cigarettes per day and BTG participants smoking an average of 9.20 (SD 7.42) cigarettes per day. Regarding smoking cessation medication, 10 of 15 participants in the DTG (67%) and 8 of 15 participants in the BTG (53%) were using bupropion at baseline. Regarding the type of tobacco used, among the 15 participants of the DTG, 8 participants (53%) used manufactured cigarettes, 2 participants (13%) used heat-not-burn tobacco, 1 participant (7%) used liquid e-cigarettes, and 4 participants (27%) used multiple tobacco products. Among the 15 participants in the BTG, 13 participants (87%) had a higher proportion of manufactured cigarette use compared than the DTG. No participants used heat-not-burn tobacco, 1 participant (1/15, 7%) used liquid e-cigarettes, and 1 participant (1/15, 7%) used multiple tobacco products. The mean FTND scores were 2.80 (SD 2.48) in the DTG and 4.87 (SD 2.62) in the BTG. Exhaled carbon monoxide levels averaged 6.13 (SD 6.21) ppm in the DTG and 6.60 (SD 4.63) ppm in the BTG. Salivary cotinine test results were positive for 11 participants (73%) and negative for 4 participants (27%) of 15 participants in the DTG, whereas 14 participants (93%) tested positive and 1 participant (7%) tested negative out of 15 participants in the DTG.

**Table 2:** Demographics and baseline characteristics of the participants (N=30).

Characteristics		DTG^a^ (n=15)	BTG^b^ (n=15)
Age (years), mean (SD)		43.07 (12.15)	48.67 (14.53)
**Sex, n (%)**			
	Male	11 (73)	12 (80)
	Female	4 (26)	3 (20)
**Education level, n (%)**			
	Elementary school graduate	0 (0)	1 (7)
	High school graduate	4 (27)	2 (13)
	Two-year college degree graduate	0 (0)	1 (7)
	Bachelor’s degree	9 (60)	9 (60)
	Graduate degree	2 (13)	2 (13)
Length of smoking (years), mean (SD)		23.32 (11.22)	29.21 (12.90)
Number of cigarettes per day, mean (SD)		9.53 (8.16)	9.20 (7.42)
**Medication for smoking cessation, n (%)**			
	Bupropion	10 (67)	8 (53)
	None	5 (33)	7 (47)
**Type of tobacco used, n (%)**			
	Manufactured cigarettes	8 (53)	13 (87)
	Heat-not-burn tobacco	2 (13)	0 (0)
	Liquid e-cigarettes	1 (7)	1 (7)
	Multiple tobacco product use	4 (27)	1 (7)
FTND^c^, mean (SD)		2.80 (2.48)	4.87 (2.62)
Exhaled carbon monoxide levels, mean (SD)		6.13 (6.21)	6.60 (4.63)
**Salivary cotinine qualitative test results, n (%)**			
	Positive	11 (73)	14 (93)
	Negative	4 (27)	1 (7)
Dropouts, n (%)		6 (40)	3 (20)

^a^DTG: digital therapeutic group.

^b^BTG: basic treatment group.

^c^FTND: Fagerström Test for Nicotine Dependence.

### Primary Efficacy Indicators

Initially, the ITT analysis was performed to assess the primary efficacy outcome. At the end of the 12-week study period, 6 of 15 participants (40%) in both the DTG and BTG reported achieving 7-day PPA. Moreover, there were no significant differences between the 2 groups at any time point (week 0: *U*=97.50, *P*=.37; week 4: *U*=90.00, *P*=.27; week 8: *U*=112.50, *P*≥.99; week 12: *U*=112.50, *P*≥.99). The mean number of smoking days over a 7-day period at the 12-week mark was 3.27 (SD 3.31) days for the DTG and 3.67 (SD 3.33) days for the BTG, with no statistically significant difference observed. In addition, the average number of cigarettes smoked over 7 days at the 12-week mark was 36.77 (SD 56.73; range 0-168) cigarettes for the DTG and 28.53 (SD 41.91; range 0-140) cigarettes for the BTG; however, this difference was not significant.

After analyzing the difference in 7-day point abstinence between baseline and each time point after the DTx program in the DTG, there was a statistically significant difference only at the 8-week point (*z*=–2.00, negative ranks=0, positive ranks=4, *P*=.046) confirming the efficacy of the DTx program (Table 3). At the 12-week point, the difference before and after the intervention within the DTG was not statistically significant but showed an estimated value relatively close to the significance level (*z*=–1.73, negative ranks=0, positive ranks=3, *P*=.08). On the other hand, in the BTG, none of the differences between the baseline and each time point after basic treatment were statistically significant at any time point.

**Figure 3:**
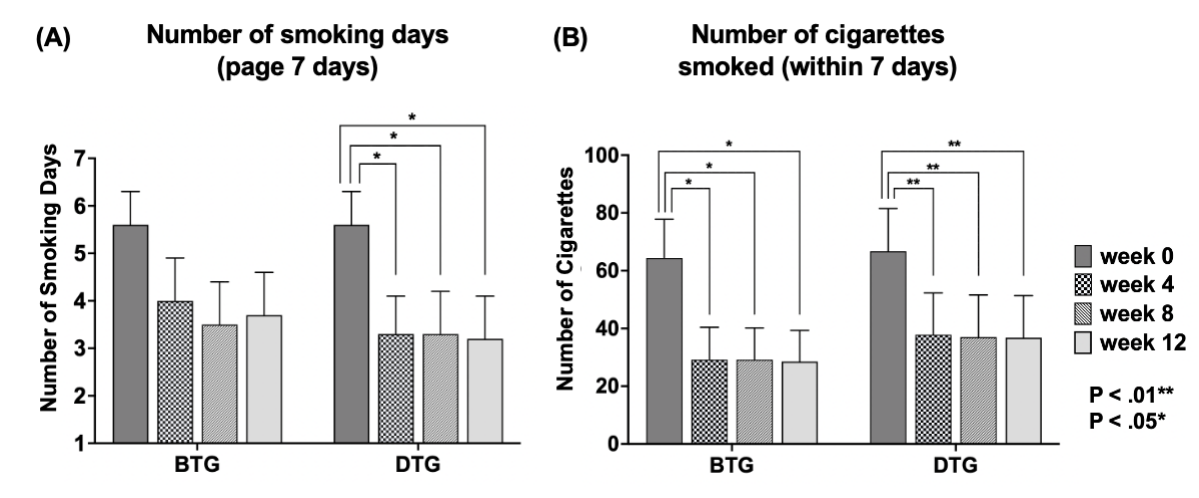
Changes in (A) and (B) over time in the digital therapeutic group (DTG) and basic treatment group (BTG).

More specifically, changes from baseline in the number of smoking days and the quantity of cigarettes smoked over a 7-day period were analyzed within each group at each time point (Figure 3, Tables 4 and 5). Regarding the number of smoking days over a 7-day period, the DTG demonstrated statistically significant reductions at all post-treatment time points compared with baseline (Figure 3, Table 4), whereas no significant changes were observed in the BTG (Figure 3, Table 4).

**Table 3:** Intention-to-treat analysis of the difference in 7-day and 30-day point prevalence abstinence before and after treatment, by assigned group.

Evaluation time points, by group				Negative ranks^a^, n (mean ranks)		Positive ranks^b^, n (mean ranks)		^Ties, n^		*z*-score		*P* value
**7-day point prevalence abstinence**												
	**BTG^c^** **(n=1** **5** **)**											
		Week4-week0	0 (0.00)		3 (2.00)		12		–1.73		.08	
		Week8-week0	1 (2.50)		3 (2.50)		11		–1.00		.32	
		Week12-week0	1 (2.00)		2 (2.00)		12		–0.58		.56	
	**DTG^d^** **(n=** **15** **)**											
		Week4-week0	0 (0.00)		2 (1.50)		13		–1.41		.16	
		Week8-week0	0 (0.00)		4 (2.50)		11		–2.00		.046	
		Week12-week0	0 (0.00)		3 (2.00)		12		–1.73		.08	
**30-day point prevalence abstinence ^e^**												
	**BTG (n=1** **5** **)**											
		Week4-week0	1 (1.50)		1 (1.50)		13		0.00		≥.99	
		Week8-week0	1 (2.50)		3 (2.50)		11		–1.00		.32	
		Week12-week0	1 (1.50)		1 (1.50)		13		0.00		≥.99	
	**DTG (n=** **15** **)**											
		Week4-week0	0 (0.00)		1 (1.00)		14		–1.00		.32	
		Week8-week0	0 (0.00)		4 (2.50)		11		–2.00		.046	
		Week12-week0	0 (0.00)		3 (2.00)		12		–1.73		.08	

^a^Smoking (0) > smoking abstinence (1).

^b^Smoking (0) < smoking abstinence (1).

^c^BTG: basic treatment group.

^d^DTG: digital therapeutic group.

^e^The 30-day point prevalence abstinence (PPA) baseline (week 0) was replaced with the 7-day PPA.

**Table 4:** Intention-to-treat analysis of the difference in the number of smoking days within 7 days before and after treatment, by assigned group.

Evaluation time points, by group			Negative ranks^a^, n (mean ranks)		Positive ranks^b^, n (mean ranks)		Ties, n		*z*-score		*P* value
**BTG^c^** **(n=1** **5** **)**											
	Week4-week0	0 (0.00)		4 (2.50)		11		–1.89		.06	
	Week8-week0	1 (1.50)		5 (3.90)		9		–1.91		.06	
	Week12-week0	1 (1.00)		4 (3.50)		10		–1.76		.08	
**DTG^d^** **(n=** **15** **)**											
	Week4-week0	0 (0.00)		6 (3.50)		9		–2.27		.02	
	Week8-week0	0 (0.00)		7 (4.00)		8		–2.41		.02	
	Week12-week0	0 (0.00)		7 (4.00)		8		–2.40		.02	

^a^Baseline number of smoking days > number of smoking days in weeks 4, 8, and 12.

^b^Baseline number of smoking days < number of smoking days in weeks 4, 8, and 12.

^c^BTG: basic treatment group.

^d^DTG: digital therapeutic group.

**Table 5:** Intention-to-treat analysis of the difference in the number of cigarettes smoked within 7 days before and after treatment, by assigned group.

Evaluation time points, by group			Negative ranks^a^, n (mean ranks)		Positive ranks^b^, n (mean ranks)		^Ties, n^		*z*-score		*P* value
**BTG^c^** **(n=1** **5** **)**											
	Week4-week0	0 (0.00)		8 (4.50)		7		–2.52		.01	
	Week8-week0	1 (1.00)		8 (5.50)		6		–2.55		.01	
	Week12-week0	2 (2.50)		8 (6.25)		5		–2.30		.02	
**DTG^d^** **(n=** **15** **)**											
	Week4-week0	0 (0.00)		8 (4.50)		7		–2.52		.01	
	Week8-week0	0 (0.00)		8 (4.50)		7		–2.53		.01	
	Week12-week0	0 (0.00)		8 (4.50)		7		–2.52		.01	

^a^Baseline number of smoking days > number of smoking days in weeks 4, 8, and 12.

^b^Baseline number of smoking days < number of smoking days in weeks 4, 8, and 12.

^c^BTG: basic treatment group.

^d^DTG: digital therapeutic group.

Both the DTG and BTG demonstrated statistically significant reductions in the number of cigarettes smoked within 7 days at all time points (Figure 3, Table 5; all *P*<.05). At the 12-week mark, the 30-day PPA rate was 33% (5/15) in the DTG and 27% (4/15) in the BTG, with no significant group differences at any of the time points (week 4: *U*=105.00, *P*=.67; week 8: *U*=112.50, *P≥*.99; week 12: *U*=120.00, *P*=.70). Additionally, since the number of smoking days and the quantity of cigarettes smoked over a 30-day period were not assessed at baseline, the results were examined using a PP analysis. The mean number of smoking days over a 30-day period at the 12-week mark was 1.89 (SD 2.85) days for the DTG and 11.67 (SD 13.19) days for the BTG, with no statistically significant difference observed. In addition, the average number of cigarettes smoked over 30 days at the 12-week mark was 3.89 (SD 6.45; range 0-16) cigarettes for the DTG and 72.63 (SD 97.94; range 0-300) cigarettes for the BTG, even though this difference in average number of cigarettes smoked between the 2 groups was not statistically significant.

Similar to the 7-day PPA, the 30-day PPA in the DTG also showed statistically significant differences at evaluation points after 8 weeks of the DTx program compared with baseline (Table 3; *z*=–2.00, negative ranks=0, positive ranks=4, *P*=.046). Additionally, at the 12-week point, the difference before and after the intervention in the 30-day PPA in the DTG was not statistically significant but showed an estimated value relatively close to the significance level (*z*=–1.73, negative ranks=0, positive ranks=3, *P*=.08). This indicates that the DTx program was effective at achieving not only 7-day smoking abstinence but also 30-day smoking abstinence. On the other hand, in the BTG, none of the differences between the baseline and each time point after basic treatment were statistically significant.

### Consistency Between Self-Reported Results and Biological Test Results

Of all 21 participants, the discordance rate between the qualitative saliva cotinine test results and the patients’ verbal self-reports was 13% (n=4) at week 4, 3% (n=1) at week 8, and 3% (n=1) at week 12. When examining the discordance rates, no significant differences were observed between the groups across the weeks. At the 4-week assessment, false negatives included participants who reported smoking over the past 30 days but reported abstinence in the past 7 days. Specifically, their exhaled carbon monoxide levels were all found to be 0 ppm or 1 ppm, indicating a nonsmoker level (≤7 ppm). On average, these false-negative participants had smoked for 6 (range 1-12) days in the past 30 days, with an average daily consumption of 5 (range 1.5-15) cigarettes. In cases of false negatives, participants reported exposure to secondhand smoke or were likely owing to a small amount of smoking at least 7 days prior. Their exhaled carbon monoxide levels were 5 ppm and 7 ppm, respectively, corresponding to nonsmoker levels. Overall, the concordance rate between self-reports and the saliva cotinine qualitative test was approximately 86% to 97%, indicating a high level of reliability for the self-reported data. Detailed concordance rates at each assessment time point are presented in Multimedia Appendix 5.

### Secondary Efficacy Indicators

**Figure 4:**
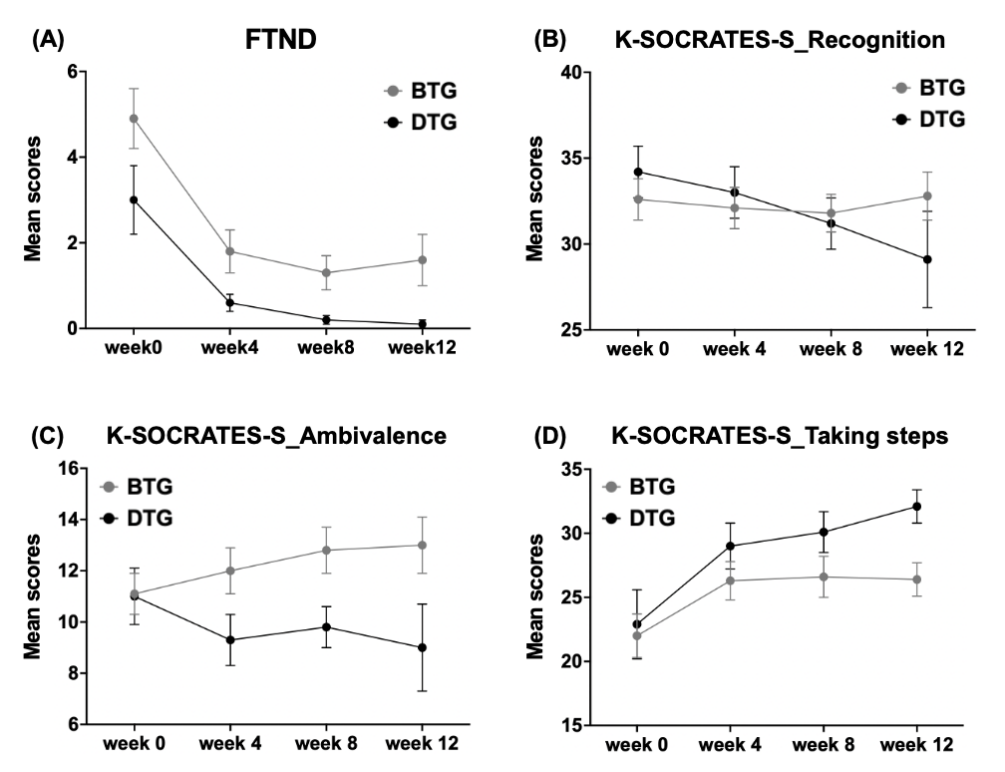
Changes in nicotine dependence and motivation for change over time in the digital therapeutic group (DTG) and basic treatment group (BTG) as shown in (A)–(D).

To evaluate the treatment effect, a PP analysis was conducted for the secondary efficacy outcomes. The FTND analysis indicated no statistically significant differences between the groups prior to treatment, and a similar pattern was observed following the program, with the results remaining comparable to those observed before treatment (Figure 4, Table 6).

The results of comparing the subfactor scores of the K-SOCRATES-S to examine the motivation to change smoking cessation between the DTG and BTG are shown in Figure 4 and Table 6. There was no significant difference in motivation recognition between the DTG and BTG owing to the random assignment (adjusted *P* values: week 0: *P=*.71; week 4: *P*=.77; week 8: *P=*.77; week 12: *P*=.71). Although no statistically significant difference was observed in motivation-ambivalence, a trend approaching significance was observed from week 4 (*U*=28.50; DTG=9, BTG=12; adjusted *P*=.09) to week 12 (*U*=28.50; DTG=9, BTG=12; adjusted *P*=.09), with differences nearing meaningful levels compared with baseline. Specifically, BTG participants consistently maintained ambivalence levels slightly higher than their baseline, whereas DTG participants tended to exhibit slightly lower levels of ambivalence compared with their baseline. Furthermore, there was a significant group difference at 12 weeks (*U*=19.00; DTG n=9, BTG n=12; adjusted *P*=.048) to the motivation-taking steps.

**Table 6:** Per-protocol comparative analysis results of supplementary indicators between the digital therapeutic group (DTG) and basic treatment group (BTG).

Assessment by week and group						Mean rank		Sum of ranks		*U*			*P* value			Adjusted *P* value^a^	
**FTND^b^**																	
	**Week 0**										30.50			.09			.09
				BTG (n=12)	12.96		155.50										
				DTG (n=9)	8.39		75.50										
	**Week 4**										29.00			.05			.07
				BTG (n=12)	13.08		157.00										
				DTG (n=9)	8.22		74.00										
	**Week 8**										26.00			.03			.07
				BTG (n=12)	13.33		160.00										
				DTG (n=9)	7.89		71.00										
	**Week 12**										31.00			.05			.07
				BTG (n=12)	12.92		155.00										
				DTG (n=9)	8.44		76.00										
**K-SOCRATES-S** ^c^ **-recognition**																	
		**Week 0**									41.00			.35			.71
			BTG (n=12)		9.92		119.00										
			DTG (n=9)		12.44		112.00										
		**Week 4**									50.00			.77			.77
			BTG (n=12)		10.67		128.00										
			DTG (n=9)		11.44		103.00										
		**Week 8**									49.50			.75			.77
			BTG (n=12)		11.38		136.50										
			DTG (n=9)		10.50		94.50										
		**Week 12**									37.50			.24			.71
			BTG (n=12)		12.38		148.50										
			DTG (n=9)		9.17		82.50										
**K-SOCRATES-S ^c^** **-a** **mbivalence**																	
		**Week 0**									45.00			.51			.51
			BTG (n=12)		11.75		141.00										
			DTG (n=9)		10.00		90.00										
		**Week 4**									28.50			.07			.09
			BTG (n=12)		13.13		157.50										
			DTG (n=9)		8.17		73.50										
		**Week 8**									23.50			.03			.09
			BTG (n=12)		13.54		162.50										
			DTG (n=9)		7.61		68.50										
		**Week 12**									28.50			.07			.09
			BTG (n=12)		13.13		157.50										
			DTG (n=9)		8.17		73.50										
**K-SOCRATES-S-t** **aking steps**																	
		**Week 0**									48.50			.70			.70
			BTG (n=12)		10.54		126.50										
			DTG (n=9)		11.61		104.50										
		**Week 4**									38.00			.25			.34
			BTG (n=12)		9.67		116.00										
			DTG (n=9)		12.78		115.00										
		**Week 8**									35.50			.19			.34
			BTG (n=12)		15.70		235.50										
			DTG (n=9)		15.30		229.50										
		**Week 12**									19.00			.01			.048
			BTG (n=12)		14.47		217.00										
			DTG (n=9)		16.53		248.00										

^a^Adjusted using the Benjamini-Hochberg method.

^b^FTND: Fagerström Test for Nicotine Dependence.

^c^K-SOCRATES-S: Korean Stages of Change Readiness and Treatment Eagerness Scale-Smoking.

## Discussion

### Principal Findings

This exploratory clinical trial aimed to evaluate the safety and preliminary efficacy of NICO-THERA, a DTx software program developed to alleviate nicotine dependence symptoms in patients diagnosed with nicotine use disorder. 

Nicotine dependence is a chronic condition with a high relapse rate necessitating long-term and consistent therapeutic management. Traditional treatments for nicotine addiction typically involve medication and face-to-face CBT sessions. However, adherence to prescribed medications is often suboptimal, which reduces the efficacy of pharmacotherapy. Additionally, CBT sessions require considerable time and financial investment from the patients, which can be a significant barrier. In practice, the national smoking cessation support services in South Korea are typically available only during working hours, which limits physical and temporal accessibility. Although available, online services are typically limited to one-time consultations via telephone or messaging. Consequently, many smokers struggle to maintain their motivation and commitment to quit outside the clinical settings in their daily lives. The number of smokers utilizing national smoking cessation services in South Korea is steadily declining, with only approximately 30% completing the program [67,68]. These challenges highlight the need for alternative strategies to address the limitations of traditional smoking cessation treatments in the digital age.

In this context, DTx, such as NICO-THERA, could play a pivotal role as an adjunct to existing smoking cessation treatments. Specifically, these interventions can be integrated into daily life by providing continuous support, along with national smoking cessation programs as a complementary method. As a digital therapy based on MET and CBT, NICO-THERA has the potential to enhance treatment accessibility for nicotine addiction and advance patient-centered participatory medicine. It can also assist health care providers with monitoring patients’ condition daily and incorporating this information into treatment plans. In this study, both the DTG and BTG participants followed identical outpatient visit schedules and pharmacotherapy regimens. However, differences in the interventions implemented in each group resulted in varying adherence rates, which may have contributed to observed differences in smoking cessation outcomes between the 2 groups. In the DTG, relatively favorable treatment adherence was observed among participants who completed the program, and no adverse device effects were reported. These findings suggest that NICO-THERA can be regarded as a safe medical device with the potential to contribute to smoking cessation. Several previous studies reported on the effectiveness of pharmacotherapy or behavioral interventions for smoking cessation, emphasizing the combination of both as clinical guidelines for successfully providing smoking cessation treatment [10-13,69]. In fact, a meta-analysis reported that combining pharmacotherapy with intensive behavioral support significantly increases the likelihood of successful smoking cessation compared with providing simple advice or support [13,58]. This suggests that NICO-THERA, as a digital therapy, has the potential to enhance the effectiveness of smoking cessation treatments by supplementing pharmacotherapy and counseling.

NICO-THERA is a 12-week DTx that systematically integrates a mobile app and VR to promote smoking cessation based on the stages of change model [55]. In the DTx program, the participants engaged in VR content for relaxation training, craving coping training, and refusal training in clinical settings. Additionally, they undertook the smoking cessation program, including cognitive restructuring, refusal skills, and coping with negative emotions through the app. Regarding primary efficacy, the 7-day and 30-day PPA of the DTG significantly improved at week 8. Additionally, significant reductions in both the number of smoking days and the number of cigarettes smoked were observed at all time points compared with baseline in the DTG. In the BTG, despite a significant reduction in cigarette consumption at weeks 4, 8, and 12, no statistically significant improvements were found in the number of smoking days and in abstinence rates. Although the group differences at each 4-week interval were not statistically significant due to the small sample size, the improvement in abstinence observed in the DTG group may be attributable to the DTx program. In this study, we identified a series of positive behavioral changes among DTG participants—including increased motivation to quit smoking—that aligned with their learning of craving management skills and strategies for overcoming cognitive distortions from week 4 to week 12. Also, from week 4 to week 12, no significant differences in nicotine dependence were observed between the DTG and BTG participants, with levels remaining comparable before and after treatment. However, the DTG exhibited a consistently significant reduction in both the number of smoking days and the number of cigarettes smoked, compared with the BTG. Therefore, by acquiring and repeatedly practicing cognitive, behavioral, and emotional coping strategies conducive to smoking cessation, participants were able to establish and consolidate resources to effectively apply coping skills. The DTx intervention may have contributed to maintaining the stability of both physical and psychological dependence on nicotine, thereby preventing its deterioration and ultimately supporting a reduction in nicotine consumption.

The progressive decline in ambivalence from week 4 and the significant increase in taking steps at week 12 suggest that the DTG participants exhibited a progressive change in their motivation to quit smoking. The initial 1 to 4 weeks of the DTx program included a series of content focused on MET approaches, such as addiction education, addressing ambivalent emotions, creating supportive environments, and understanding confidence in quitting smoking. From week 4, they demonstrated an improved ability to resolve ambivalence and strengthen their commitment to change, thereby enhancing their motivation. By week 12, DTG participants had solidified their resolve to sustain smoking cessation, further demonstrating the effectiveness of the program’s approach. Notably, at week 8, the DTG exhibited a significant improvement in the primary efficacy outcome, abstinence, compared with baseline, with this effect being relatively sustained through week 12. In the 8 to 12 weeks of the program, the emphasis shifted to consolidating and maintaining the behavioral skills acquired during earlier phases, with a focus on training participants to manage not only cravings but also emotional regulation strategies. These strategies aim to address factors such as depression, anxiety, and anger, which may influence smoking behavior. As a result, the impact of the intervention may have become most evident by week 8, with the remainder of the program serving to reinforce and sustain these changes. This pattern is consistent with those found in previous studies that demonstrate a progression of motivation and behavioral change from the precontemplation stage to the contemplation stage then to the preparation stage and finally the action stage [55-57]. In contrast, the BTG maintained a similar level of recognition, whereas the scores for ambivalence and taking steps gradually increased. This pattern suggests that the BTG remained at the precontemplation stage and contemplation stage without fully transitioning to the action stage, likely owing to unresolved ambivalence and ongoing conflict between the cognitive and behavioral dimensions [57]. Smoking cessation is not merely a matter of short-term efforts; it requires ongoing management to ensure long-term success. NICO-THERA, as a software that combines app and VR components, provides cognitive, behavioral, and emotional strategies designed to support smoking cessation across various stages of behavioral change. Notably, VR allows participants to engage in motivational, cognitive, and behavioral training in clinical settings, helping them develop strategies to cope with smoking triggers. The smartphone app supports self-directed therapy and habit formation in daily life through its content and daily functions. As a digital therapy based on MET and CBT, NICO-THERA has the potential to enhance the effectiveness of smoking cessation treatments by expanding combined DTx interventions and improving accessibility.

In summary, the results demonstrated that adults who participated in NICO-THERA had a significantly higher success rate for smoking abstinence. Additionally, compared with those who received basic treatment, participants in the DTx program exhibited higher motivation to quit smoking. Participants in the DTx program exhibited adaptive changes conducive to smoking cessation consistent with the weekly intervention strategies of the MET and CBT. Thus, the efficacy of the DTx program was validated, suggesting its greater potential to facilitate successful smoking cessation than basic treatment.

### Limitations

This study has some limitations. As a pilot study, the sample size was kept small, limiting the statistical power to detect significant differences in some analyses.

The small sample size (30 participants) necessitated the use of nonparametric tests, which affected the generalizability of the results. In addition, there were limitations in analyzing the impact of medication and counseling adherence on the efficacy of DTx. Future studies with larger sample sizes are required to confirm the observed trends and to establish statistical significance through parametric tests. Additionally, the open-label nature of the study, in which both participants and researchers were aware of group assignments, may have introduced bias in outcome reporting.

The DTG had a higher dropout rate than the BTG. Specifically, 5 participants in the DTG dropped out in week 4; notably, 3 of them expressed a willingness to continue but were unable to visit the hospital due to scheduling issues. NICO-THERA not only required in-person VR sessions but also involved relatively greater time demands for device setup and execution compared with the passive activity of video viewing. Therefore, limited device and temporal accessibility may have contributed to the higher dropout rate. In the future, developing portable or home-based VR devices could help mitigate this limitation by enabling patients to receive treatment without being restricted by the temporal constraints of hospital visits.

Future directions for VR in smoking cessation may involve more personalized VR experiences that adapt to users’ physiological and psychological states in real time, potentially through the integration of biofeedback mechanisms. As VR technology continues to evolve, such personalized interventions could allow users to engage in dynamically responsive therapeutic environments that match their stress levels, craving intensity, or motivational readiness.

In this study, NICO-THERA enabled a degree of personalization by allowing participants to choose among VR scenarios tailored for relaxation, craving coping, and refusal training. This scenario selection was based on the user’s preferences or situational relevance, offering flexibility within the intervention. However, the program did not yet incorporate adaptive customization based on the participants’ stage of change or real-time physiological input. We acknowledge this as a limitation and propose it as a valuable direction for future development.

Building on this pilot study, future confirmatory trials should aim to address the limitations identified herein, particularly those related to accessibility and personalization. By refining the VR delivery method and enhancing the adaptability of mobile and VR content, the NICO-THERA intervention may further improve its clinical efficacy and user engagement.

### Conclusion

We identified the preliminary safety and efficacy of NICO-THERA, a DTx combining MET and CBT that has the potential to improve smoking cessation outcomes. By incorporating CBT and MET content into a sequenced digital format and linking each weekly module with a corresponding VR session, NICO-THERA offers a distinctive treatment flow that contrasts with mobile-only interventions primarily focused on information delivery or tracking tools. Participants in the DTG experienced significant reductions in nicotine dependence and maintained a stronger practical motivation for quitting smoking. These results suggest that integrating immersive behavioral training with structured cognitive strategies may enhance engagement and support core behavioral change in smoking cessation. It has been suggested that NICO-THERA may serve as a valuable complement to traditional pharmacotherapy and counseling in smoking cessation programs. Although the statistical significance of the group differences in smoking cessation rates could not be confirmed due to the limitations of the sample size, the smoking cessation rate of the DTG group increased significantly at week 8, as compared with baseline. Future research should confirm these findings in larger clinical trials and explore the long-term efficacy of integrating VR-based interventions into smoking cessation programs. Additionally, studies should assess their scalability across diverse clinical and community settings.

## Appendix

Multimedia Appendix 1 Background settings for image relaxation training through virtual reality (VR), presented sequentially: forest, campfire, beach.

Multimedia Appendix 2 Virtual reality (VR)-based training session for craving coping: A concept image from the storyboard of the VR content illustrating a situation at a social gathering where friends are stepping out to smoke.

Multimedia Appendix 3 Virtual reality (VR)-based training session for refusal training: A concept image from the storyboard of the VR content depicting a situation where a friend offers a cigarette to someone who is in the process of quitting smoking.

Multimedia Appendix 4 App screenshots of the digital therapeutic intervention NICO-THERA, presented sequentially from left to right: Smoking cessation therapy contents, Smoking cessation diary and medication log, and a graph displaying the number of cigarettes smoked and changes in motivation levels.

Multimedia Appendix 5 Concordance rate between self-reported and saliva cotinine qualitative test results.

Multimedia Appendix 6 CONSORT-eHEALTH checklist (V 1.6.1).

## Abbreviations

BTG: basic treatment group
CBT: cognitive behavioral therapy
DSM-5: Diagnostic and Statistical Manual of Mental Disorders, Fifth Edition
DTG: digital therapeutic group
DTx: digital therapeutic
FTND: Fagerström Test for Nicotine Dependence
ITT: intention to treat
K-SOCRATES-S: Korean Stages of Change Readiness and Treatment Eagerness Scale-Smoking
MET: motivational enhancement therapy
mHealth: mobile health
OECD: Organisation for Economic Co-operation and Development
PP: per-protocol
PPA: point prevalence abstinence
RCT: randomized controlled trial
SSQ: Simulator Sickness Questionnaire
VR: virtual reality
