# Supplementary figures and images for: Pilot Study of a Mobile, Virtual Reality–Based Digital Therapeutic for Smoking Cessation: Randomized Controlled Trial
Source: JMIR Mhealth Uhealth. 2025 Nov 12;13:e66411. doi: 10.2196/66411 (PMC12658401; doi:10.2196/66411)

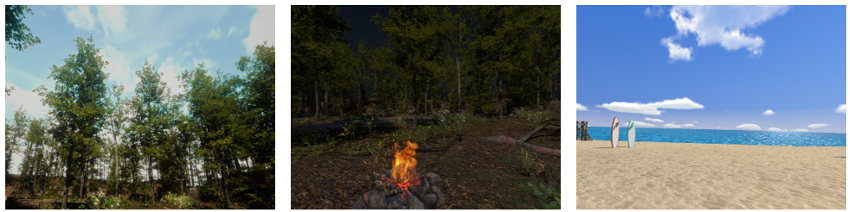

Supplement: Multimedia Appendix 1 [file mhealth_v13i1e66411_app1.png]

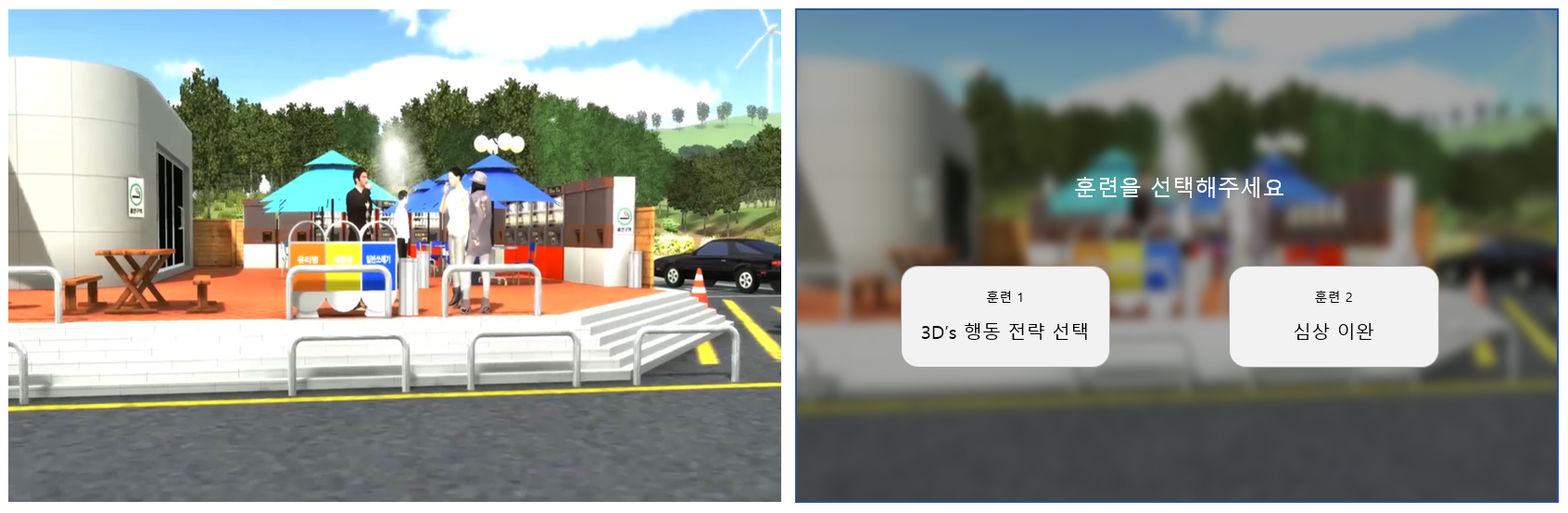

Supplement: Multimedia Appendix 2 [file mhealth_v13i1e66411_app2.png]

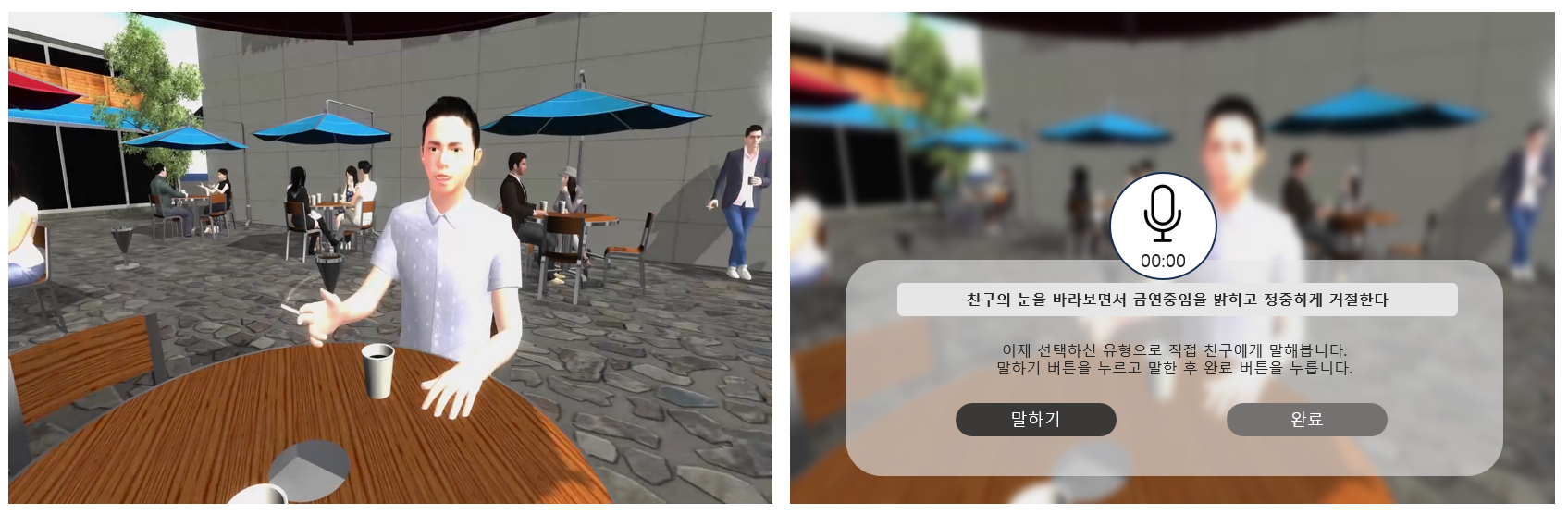

Supplement: Multimedia Appendix 3 [file mhealth_v13i1e66411_app3.png]

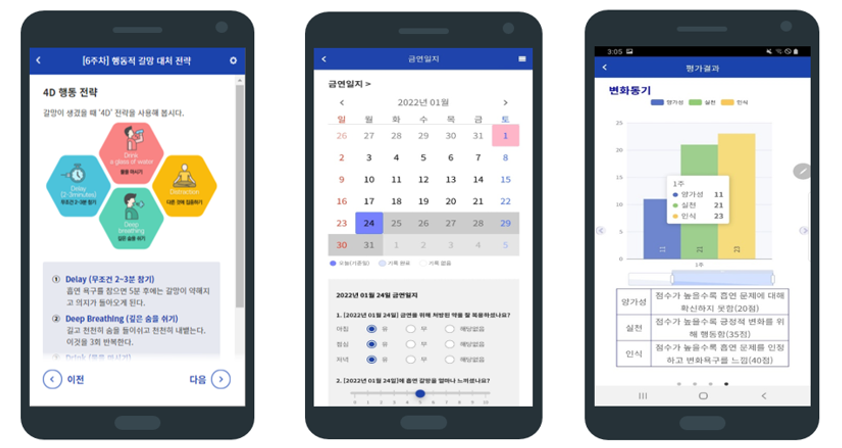

Supplement: Multimedia Appendix 4 [file mhealth_v13i1e66411_app4.png]
