# Supplementary material for: Pilot Study of a Mobile, Virtual Reality–Based Digital Therapeutic for Smoking Cessation: Randomized Controlled Trial
Source: JMIR Mhealth Uhealth. 2025 Nov 12;13:e66411. doi: 10.2196/66411 (PMC12658401; doi:10.2196/66411)
Supplement: Multimedia Appendix 5 [file mhealth_v13i1e66411_app5.docx]

**Multimedia Appendix 5.** Concordance rate between self-reported and saliva cotinine qualitative test results *N*=30

| Classification of diagnosis  for smoking abstinence/smoking | | | Biological Test Results  : Saliva cotinine qualitative test [%(n)] | | |
| --- | --- | --- | --- | --- | --- |
|  |  |  | S | SA | All |
| Self-Reported Results: Verbal Reports from Participants  (30-day point prevalence abstinence) | week 4 | S | true positive  63 (19) | false negative  13 (4) | 76 (23) |
|  |  | SA | false positive  0 (0) | true negative  23 (7) | 23 (7) |
|  |  | All | 63 (19) | 37 (11) | 100 (30) |
|  | week 8 | S | true positive  60 (18) | false negative  0 (0) | 60 (18) |
|  |  | SA | false positive  3 (1) | true negative  37 (11) | 40 (12) |
|  |  | All | 63 (19) | 37 (11) | 100 (30) |
|  | week 12 | S | true positive  67 (20) | false negative  0 (0) | 67 (20) |
|  |  | SA | false positive  3 (1) | true negative  30 (9) | 33 (10) |
|  |  | All | 70 (21) | 30 (9) | 100 (30) |

Note. S: Smoking, SA: Smoking Abstinence
